# Reconstitution of prenyltransferase activity on nanodiscs by components of the rubber synthesis machinery of the Para rubber tree and guayule

**DOI:** 10.1038/s41598-022-07564-y

**Published:** 2022-03-08

**Authors:** Fu Kuroiwa, Akira Nishino, Yasuko Mandal, Masataka Honzawa, Miki Suenaga-Hiromori, Kakeru Suzuki, Yukie Takani, Yukino Miyagi-Inoue, Haruhiko Yamaguchi, Satoshi Yamashita, Seiji Takahashi, Yuzuru Tozawa

**Affiliations:** 1 Graduate School of Science and Engineering, Saitama University, 255 Shimo-Okubo, Sakura-ku, Saitama, Saitama 338-8570 Japan; 2 Graduate School of Engineering, Tohoku University, Sendai, Miyagi 980-8579 Japan; 3 Sumitomo Rubber Industries Ltd, Kobe, Hyogo 651-0072 Japan; 4 Graduate School of Natural Science and Technology, Kanazawa University, Kakuma, Kanazawa 920-1192 Japan

**Keywords:** Biochemistry, Plant sciences

## Abstract

Natural rubber of the Para rubber tree (*Hevea brasiliensis*) is synthesized as a result of prenyltransferase activity. The proteins HRT1, HRT2, and HRBP have been identified as candidate components of the rubber biosynthetic machinery. To clarify the contribution of these proteins to prenyltransferase activity, we established a cell-free translation system for nanodisc-based protein reconstitution and measured the enzyme activity of the protein-nanodisc complexes. Co-expression of HRT1 and HRBP in the presence of nanodiscs yielded marked polyisoprene synthesis activity. By contrast, neither HRT1, HRT2, or HRBP alone nor a complex of HRT2 and HRBP manifested such activity. Similar analysis of guayule (*Parthenium argentatum*) proteins revealed that three HRT1 homologs (PaCPT1–3) manifested prenyltransferase activity only when co-expressed with PaCBP, the homolog of HRBP. Our results thus indicate that two heterologous subunits form the core prenyltransferase of the rubber biosynthetic machinery. A recently developed structure modeling program predicted the structure of such heterodimer complexes including HRT1/HRBP and PaCPT2/PaCBP. HRT and PaCPT proteins were also found to possess affinity for a lipid membrane in the absence of HRBP or PaCBP, and structure modeling implicated an amphipathic α-helical domain of HRT1 and PaCPT2 in membrane binding of these proteins.

## Introduction

Natural rubber (NR) is an industrially important product whose supply has been mainly dependent on the Para rubber tree (*Hevea brasiliensis*). The unique physical properties of NR allow the manufacture of a variety of polymer products. However, the production of NR from the rubber tree is largely limited to Southeast Asia, and a stable supply of NR to support expansion of demand is not guaranteed. NR has therefore been considered a strategically important natural product in many countries. Plants such as guayule (*Parthenium argentatum*) and Russian dandelion (*Taraxacum kok-saghyz*) have been identified as potential alternative sources for the production of NR, and the NR biosynthetic systems of these species as well as of the Para rubber tree have been investigated^1–7^.

NR is an isoprenoid with a backbone structure comprising a polymerized C_5_H_8_ isoprene unit with a cis-1,4 configuration. This backbone is also present in dolichols, which function as carriers during protein glycosylation in eukaryotes. However, the mechanism underlying the marked polymerization characteristic of NR biosynthesis, which results in the production of polyisoprene with a molecular size of ~ 1 MDa, has remained to be elucidated. Our collaborative group previously isolated two cis-prenyltransferase (cPT) homologs, designated *Hevea* rubber transferase (HRT) 1 and HRT2, from *H. brasiliensis* RRIM 600 (hereafter referred to simply as *H. brasiliensis* if not indicated otherwise), with these proteins being predominantly localized to rubber particles^1^. Cell-free synthesis of HRT1 in the presence of detergent-washed rubber particles derived from *H. brasiliensis* allowed reconstitution of NR biosynthetic activity in vitro^8^. A cPT-like protein (cPTL) of *H. brasiliensis* that interacts with HRT1 and rubber elongation factor (REF)^9^ was also identified and designated HRT1-REF bridging protein (HRBP)^8^. The NR biosynthesis machinery was thus proposed to include HRT1 and HRBP, as well as the key factors REF and small rubber particle protein (SRPP)^10^.

cPTL proteins associated with rubber particles have also been identified in other NR-producing plants including lettuce (*Lactuca sativa*) and Russian dandelion, and knockdown of the genes encoding these proteins was found to reduce rubber content^4,11,12^. These in vivo findings thus implicated cPTL as a key player in NR biosynthesis. It has been reported that two heterologous subunits are required for cPT activity not only in NR-producing plants but also in mammals and archaea^13–15^. On the other hand, expression of several individual cPTs including HRT1 and HRT2 in a yeast *rer2* mutant, which is deficient in the activity of the major cPT of this species, resulted in the restoration of enzyme activity for the production of dolichol-size polyisoprenes in the absence of co-expression of cPTL^2,16–18^. Furthermore, introduction of the lettuce cPT protein LsCPT3 to washed rubber particles from *H. brasiliensis* reconstituted synthetic activity for NR-size polyisoprene even without co-expression of LsCPTL2^8^. These observations have thus questioned the notion that cPTL is essential for cPT activity.

Cell-free systems provide an effective tool for the production of membrane proteins^19,20^, and various modified such systems have been described^21,22^. A cell-free system supplemented with nanodiscs has been developed for the synthesis of *Escherichia coli* membrane proteins with functional folds^23^. Nanodiscs are disclike forms of lipid-bilayer membranes surrounded by a membrane scaffold protein (MSP)^24^. To gain insight into the core enzyme complex of the rubber biosynthetic machinery, we have now established a nanodisc-supplemented cell-free system for reconstitution of prenyltransferase activity (Supplementary Fig. S1) and have characterized the enzyme activities of purified protein-nanodisc complexes. We found that the combination of HRT1 and HRBP reconstitutes prenyltransferase activity on nanodiscs. Both HRT1 and HRBP were found to possess an affinity for lipid bilayers, and we identified the regions of these proteins likely responsible for membrane binding by structural modeling. We also show that the guayule homologs of these Para rubber tree proteins likewise reconstitute an active prenyltransferase enzyme on nanodiscs, and the prenyltransferase subunits of *H. brasiliensis* and *P. argentatum* were partially compatible. Homology modeling predicted that amino acid residues located at a putative interface between HRT1 and HRBP, or between their guayule homologs, are conserved in a human cPT/cPTL complex (DHDDS/NgBR). Our biochemical and in silico analyses suggest that direct interaction between plant cPT and cPTL proteins gives rise to prenyltransferase activity.

## Results

### Cell-free synthesis and enzyme assay for HRT1, HRT2, and HRBP proteins

**Figure 1:**
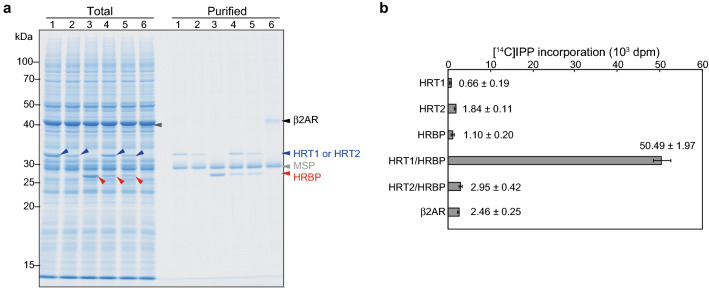
Preparation and characterization of protein-nanodisc complexes. (**a**) HRT1, HRT2, HRBP, HRT1 plus HRBP, HRT2 plus HRBP, and β2AR (lanes 1–6, respectively) were synthesized with a wheat-germ cell-free system in the presence of nanodiscs. The protein-nanodisc complexes were purified by Ni–NTA column chromatography and, together with a portion of the translation reaction mixture corresponding to 1% of the input for purification (Total), were subjected to SDS-PAGE and staining with CBB. Arrowheads indicate HRT1 or HRT2 (blue), HRBP (red), β2AR (black), and MSP (gray). The positions of molecular size markers are indicated on the left. (**b**) Polyisoprene synthesis activity assay for the purified protein-nanodisc complexes in (**a**). The incorporation of [^14^C]IPP into macromolecules extracted with 1-butanol was measured. Data are means ± s.d. from three independent experiments.

To investigate the functions of HRT1, HRT2, and HRBP, we synthesized each protein with a wheat-germ cell-free translation system. Given that HRBP is thought to function cooperatively with HRT1 or HRT2^8^, we also attempted co-translation of the combinations of HRBP and either HRT protein with the cell-free system. The human β_2_-adrenergic receptor (β2AR) was synthesized as a control^25^. The cDNAs for HRT1, HRT2, HRBP, and β2AR were cloned into the cell-free expression plasmid pYT08, and the resultant plasmids were subjected to transcription and subsequent cell-free protein synthesis in the presence of preassembled asolectin nanodiscs (Supplementary Fig. S1). The resulting protein-nanodisc complexes were isolated by immobilized metal affinity chromatography (IMAC) and analyzed by SDS–polyacrylamide gel electrophoresis (PAGE) with Coomassie brilliant blue (CBB) staining (Fig. 1a). The molecular masses of HRT1, HRT2, HRBP, β2AR, and MSP (a component of the nanodiscs) derived from their electrophoretic mobilities were essentially consistent with the predicted values of 33.1, 32.7, 29.4, 46.5, and 32.6 kDa, respectively. The results thus indicated that each synthesized protein was associated with the nanodiscs purified from the translation reaction mixtures, revealing that HRT1, HRT2, and HRBP behave as membrane proteins.

We next assessed each protein-nanodisc complex for prenyltransferase activity. The isolated complexes were thus assayed for polyisoprene synthesis activity with ^14^C-labeled isopentenyl pyrophosphate (IPP). Nanodiscs containing HRT1, HRT2, or HRBP alone or those containing both HRT2 and HRBP did not catalyze incorporation of [^14^C]IPP monomer into macromolecules extracted from the reaction mixture with 1-butanol (Fig. 1b). In contrast, the nanodiscs containing both HRT1 and HRBP mediated marked incorporation of [^14^C]IPP into macromolecules (Fig. 1b).

### Effect of sequential expression of HRT1 and HRBP on enzymatic activity

**Figure 2:**
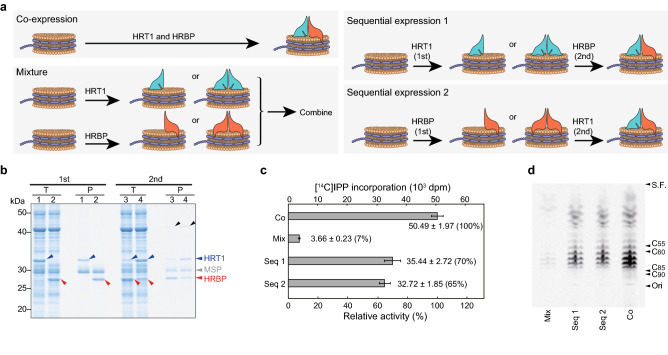
Sequential expression and characterization of HRT1/HRBP-nanodisc complexes. (**a**) Schematic representation of protein expression protocols. The mRNAs for HRT1 and HRBP were translated together in the presence of nanodiscs (Co-expression); purified HRT1-nanodisc and HRBP-nanodisc complexes were mixed (Mixture); or HRBP or HRT1 was synthesized in the presence of HRT1-nanodisc or HRBP-nanodisc complexes, respectively (Sequential expression 1 and 2, respectively). It is also possible that HRT1 and HRBP exist as homodimers on nanodiscs (see Discussion). (**b**) Purified protein-nanodisc complexes (P) as well as a portion of the translation reaction mixture corresponding to 1% of the input for purification (T) were subjected to SDS-PAGE and stained with CBB. 1st and 2nd indicate the first and second expression, respectively. Lanes 1 to 4 correspond to expression of HRT1 in the presence of empty nanodiscs, expression of HRBP in the presence of empty nanodiscs, expression of HRBP in the presence of HRT1-nanodisc complexes, and expression of HRT1 in the presence of HRBP-nanodisc complexes, respectively. Arrowheads indicate HRT1 (blue), HRBP (red), and MSP (gray) as well as a co-purified protein from the wheat-germ extract (black). (**c**) Polyisoprene synthesis activity assay for the purified protein-nanodisc complexes. Co, co-expression; Mix, mixture; Seq 1, sequential expression 1; Seq 2, sequential expression 2. The incorporation of [^14^C]IPP into macromolecules extracted with 1-butanol was measured. Data are means ± s.d. from three independent experiments. (**d**) Analysis of chain length for the ^14^C-labeled polyisoprenes extracted by 1-butanol was performed by TLC and autoradiography. The positions of the origin (Ori), solvent front (S.F.), and authentic standards are indicated on the right side.

Our results showed that co-expression of HRT1 and HRBP in the presence of nanodiscs reconstituted prenyltransferase activity in vitro. We next examined the possible effect of sequential expression of the two proteins on enzyme activity. HRT1-nanodisc and HRBP-nanodisc complexes were purified and added to the reaction mixtures for cell-free protein synthesis of HRBP and HRT1, respectively (Fig. 2a). The nanodiscs were then again purified and analyzed by SDS-PAGE (Fig. 2b), which revealed that either expression sequence resulted in the association of HRT1 and HRBP with nanodiscs. Each of the purified HRT1/HRBP-nanodisc complexes was also found to possess prenyltransferase activity in vitro (Fig. 2c). Thin-layer chromatography (TLC) analysis revealed that the patterns of polyisoprene elongation were also similar for the HRT1/HRBP-nanodisc complexes produced by co-expression or sequential expression of the two proteins (Fig. 2d). On the other hand, the simple mixture of HRT1-nanodisc and the HRBP-nanodisc complexes did not result in substantial reconstitution of prenyltransferase activity (Fig. 2c,d). These results thus suggest that the reconstitution of prenyltransferase activity requires direct contact between HRT1 and HRBP on the surface of nanodiscs, with the order of HRT1 and HRBP protein synthesis in the cell-free system not having a substantial effect on enzyme activity.

### Characterization of guayule cPT and cPTL proteins

**Figure 3:**
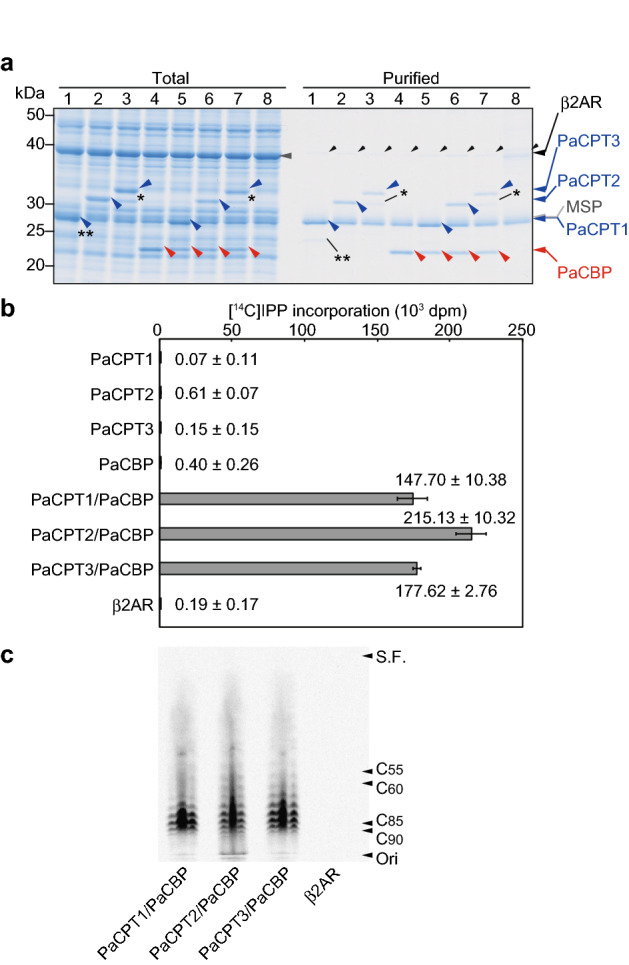
Characterization of guayule homologs of HRT1 and HRBP. (**a**) The guayule proteins PaCPT1, PaCPT2, PaCPT3, and PaCBP were synthesized with a wheat-germ cell-free system in the presence of nanodiscs either separately (lanes 1–4, respectively) or together in the combinations PaCPT1/PaCBP, PaCPT2/PaCBP, or PaCPT3/PaCBP (lanes 5–7, respectively). The protein-nanodisc complexes were then purified and analyzed by SDS-PAGE and CBB staining. β2AR was similarly synthesized and purified as a control (lane 8). A portion of each translation reaction mixture corresponding to 1% of the input for purification (Total) was also analyzed. Arrowheads indicate PaCPT1, PaCPT2, or PaCPT3 (blue); PaCBP (red); β2AR (large black); MSP (gray); or a wheat-germ protein co-purified by Ni–NTA column chromatography (small black). Single and double asterisks indicate a truncated form of PaCPT3 translated from an internal initiation codon (Supplementary Fig. S4) and an uncharacterized translation product for PaCPT1 mRNA, respectively. (**b**) Polyisoprene synthesis activity assay for the purified protein-nanodisc complexes in (**a**). The incorporation of [^14^C]IPP into macromolecules extracted with 1-butanol was measured. Data are means ± s.d. from three independent experiments. (**c**) Analysis of chain length for the ^14^C-labeled polyisoprenes extracted by 1-butanol was performed by TLC and autoradiography.

Three HRT1 homologs (PaCPT1, PaCPT2, and PaCPT3) and one HRBP homolog (PaCBP) have been identified for *P. argentatum*^6^. We next prepared protein-nanodisc complexes for these guayule proteins and assayed them for prenyltransferase activity in vitro. SDS-PAGE analysis of the purified protein-nanodisc complexes indicated that the electrophoretic mobility of PaCPT1 was similar to that of MSP (Fig. 3a and Supplementary Fig. S2b), with co-migration of the two proteins being confirmed by radioisotope labeling of PaCPT1 (Supplementary Fig. S3). The prenyltransferase activity assay (Fig. 3b) and TLC analysis (Fig. 3c) revealed that [^14^C]IPP incorporation into macromolecules extracted by 1-butanol was catalyzed by PaCPT1/PaCBP, PaCPT2/PaCBP, and PaCPT3/PaCBP complexes but not by the individual guayule proteins.

In the preparation of protein-nanodisc complexes, we observed two distinct bands for the PaCPT3 translation reaction mixture: a larger, major band and a smaller, fainter band (Fig. 3a and Supplementary Fig. S3). The mRNA sequence for PaCPT3 contains an alternative initiation codon that is located downstream of the first AUG and which corresponds to the position of the initiation codon for PaCPT2 (Supplementary Fig. S4a). We therefore constructed an expression vector for the NH_2_-terminally truncated form of PaCPT3 (PaCPT3_dN15) and prepared a PaCPT3_dN15–nanodisc complex. The electrophoretic mobility of PaCPT3_dN15 matched that of the smaller molecule observed during preparation of the PaCPT3-nanodisc complex (Supplementary Fig. S4b), and the PaCPT3_dN15/PaCBP complex manifested prenyltransferase activity similar to that of PaCPT3/PaCBP (Supplementary Fig. S4c, d). The 15 amino acids at the NH_2_-terminus of PaCPT3 therefore do not appear to affect enzymatic function.

**Figure 4:**
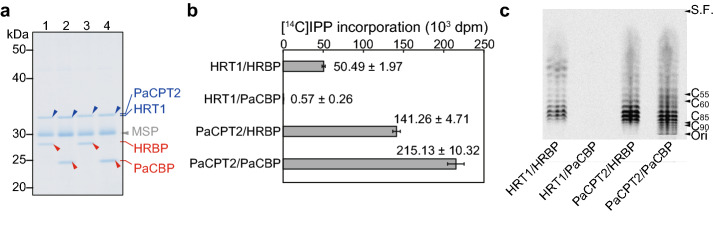
Prenyltransferase subunit compatibility between *H. brasiliensis* and *P. argentatum*. (**a**) Protein-nanodisc complexes were prepared by co-translation of HRT1 and HRBP (lane 1), HRT1 and PaCBP (lane 2), PaCPT2 and HRBP (lane 3), or PaCPT2 and PaCBP (lane 4). The purified complexes were analyzed by SDS-PAGE and CBB staining. Arrowheads indicate HRT1 or PaCPT2 (blue), HRBP or PaCBP (red), or MSP (gray). (**b**) Polyisoprene synthesis activity assay for the purified complexes in (**a**). The incorporation of [^14^C]IPP into macromolecules extracted with 1-butanol was measured. Data are means ± s.d. from three independent experiments. (**c**) Analysis of chain length for the ^14^C-labeled polyisoprenes extracted by 1-butanol was performed by TLC and autoradiography.

We investigated the interspecies compatibility of cPT catalytic subunits (HRT1 or PaCPT2) and their associated proteins (HRBP or PaCBP) with regard to the reconstitution of enzyme activity with nanodiscs (Fig. 4a). The catalytic activity of PaCPT2 was supported by HRBP, although not to the same extent as it was by PaCBP, whereas that of HRT1 was not supported by PaCBP (Fig. 4b,c). PaCPT2/PaCBP appeared to possess a greater prenyltransferase activity than HRT1/HRBP, which might account in part for the activity observed with PaCPT2/HRBP.

As indicated in Methods, the amount of each protein-nanodisc complex was set so that 0.5 µg of either cPT protein, HRT1 (approximately 302 µM as final concentration) or PaCPT2 (301 µM), is contained in each reaction mixture for the comparative enzyme assay. Estimated concentrations of accompanying cPTL proteins in the reactions were, approximately 361 µM for HRBP and 404 µM for PaCBP, respectively (Fig. 4a). As shown in Fig. 4b, higher activities of PaCPT2/PaCBP-nanodiscs than that of HRT1/HRBP-nanodiscs were constantly confirmed.

### Structure modeling of HRT1, HRBP, and their homologs

**Figure 5:**
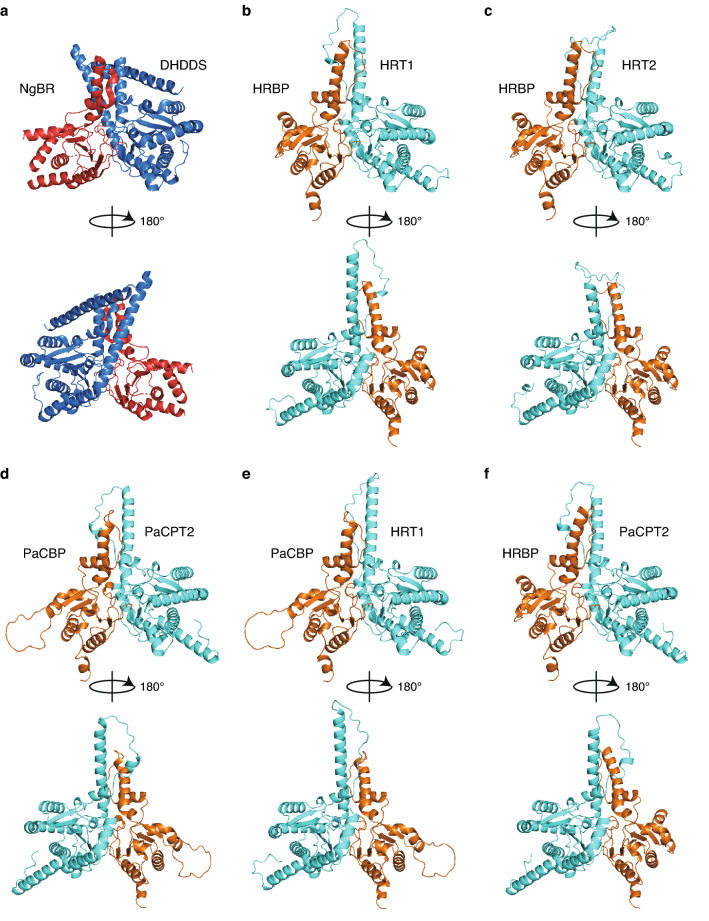
Structure models for plant cPT/cPTL complexes. (**a**) Structure of the human prenyltransferase complex DHDDS/NgBR (PDB code 6W2L). (**b**–**f**) Structural models for HRT1/HRBP, HRT2/HRBP, PaCPT2/PaCBP, HRT1/PaCBP, and PaCPT2/HRBP, respectively. Plant cPT and NH_2_-terminally truncated cPTL proteins are colored cyan and orange, respectively. Each pair of images represents views before and after vertical rotation through 180 degrees.

**Figure 6:**
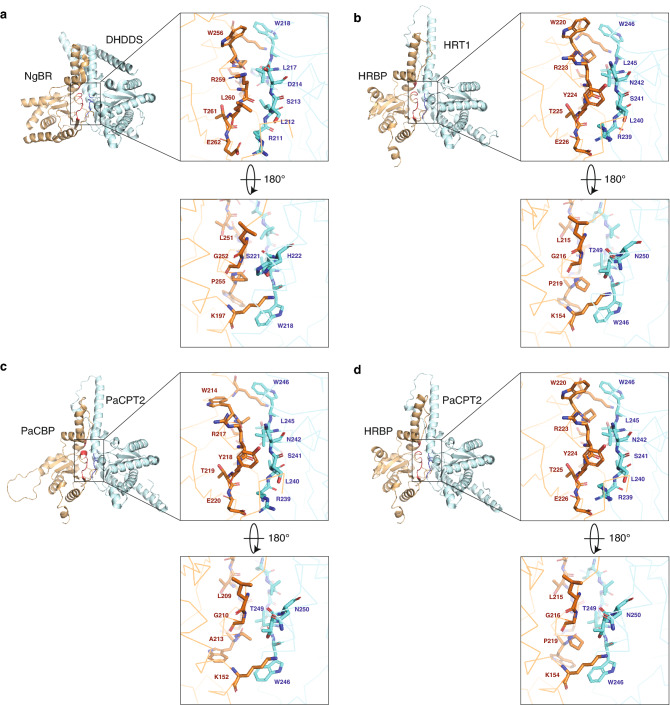
Amino acid residues at the putative interface of plant cPT/cPTL complexes. (**a**) Interface of the human prenyltransferase complex DHDDS/NgBR (PDB code 6W2L). (**b**–**d**) Putative interface regions of structural models for HRT1/HRBP, PaCPT2/PaCBP, and PaCPT2/HRBP, respectively. cPT and NH_2_-terminally truncated cPTL proteins are colored cyan and orange, respectively.

To gain insight into the structures of cPT/cPTL complexes, we obtained structure models of these proteins with a recently developed artificial intelligence system, AlphaFold2^26,27^. Crystal structures of the human cPT/cPTL complex DHDDS/NgBR have been described by two groups^28,29^. Given that these x-ray crystallography analyses were performed with a complex of full-length DHDDS and a truncated form of NgBR lacking 72 or 78 amino acids at the NH_2_-terminus, we also modeled cPTL proteins lacking the corresponding NH_2_-terminal regions (Supplementary Table S1). Structure models for the heterodimers HRT1/HRBP (Fig. 5b and Supplementary Fig. S5a) and PaCPT2/PaCBP (Fig. 5d and Supplementary Fig. S5c) resembled the structure of DHDDS/NgBR (Fig. 5a)^28,29^. The prediction for HRT2/HRBP (Fig. 5c and Supplementary Fig. S5b), which did not manifest prenyltransferase activity in vitro, was also similar to that for HRT1/HRBP. Predicted models for the interspecies complexes HRT1/PaCBP (Fig. 5e and Supplementary Fig. S5d) and PaCPT2/HRBP (Fig. 5f and Supplementary Fig. S5e) also resembled that for HRT1/HRBP, regardless of whether or not they showed prenyltransferase activity in vitro. The models showed that amino acid residues that form an interaction interface between subunits were highly conserved among cPT/cPTL pairs including DHDDS/NgBR (Fig. 6 and Supplementary Fig. S6). Resolution of the crystal structure of DHDDS/NgBR suggested the presence of an electrostatic interaction between R211 of DHDDS and E262 of NgBR as well as a hydrogen bond between D214 of DHDDS and R259 of NgBR^28^. Residues corresponding to R211 of DHDDS and E262 of NgBR are conserved in the plant cPT/cPTL models (Fig. 6 and Supplementary S6). Residues corresponding to R259 of NgBR are conserved in the plant cPTL proteins, whereas D214 of DHDDS is replaced by N in plant cPT proteins, with this N residue also possibly forming a hydrogen bond (Fig. 6 and Supplementary Fig. S6).

**Figure 7:**
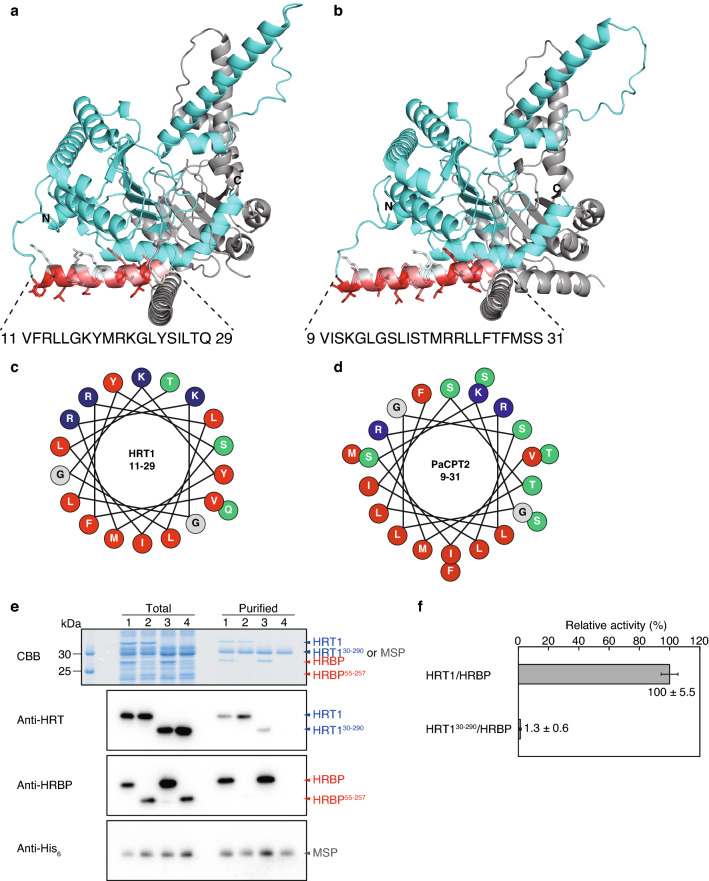
Putative membrane binding domain of plant cPT proteins. (**a**, **b**) Structural models for HRT1/HRBP (**a**) and PaCPT2/PaCBP (**b**) built from the full-length amino acid sequences (Supplementary Fig. S5). HRT1 and PaCPT2 are shown in cyan, and HRBP and PaCBP in gray, with the exception that NH_2_-terminal α-helices of HRT1 and PaCPT2 that are predicted to interact with lipid membranes are colored according to a scale from white to red depending on the hydrophobicity of the amino acid residues (with dark red indicating the greatest hydrophobicity). (**c**, **d**) Helical wheel diagrams of the NH_2_-terminal α-helices of HRT1 (**c**) and PaCPT2 (**d**). Hydrophobic, positively charged, and uncharged polar residues are indicated in red, blue, and green, respectively. (**e**) Protein-nanodisc complexes were prepared by co-translation of HRT1 and HRBP, HRT1 and HRBP(55–257), HRT1(30–290) and HRBP, or HRT1(30–290) and HRBP(55–257) (lanes 1–4, respectively). The purified complexes were subjected to SDS-PAGE followed by CBB staining or immunoblot analysis with antibodies to HRT1, to HRBP, or to His_6_ (for detection of MSP). A portion of each translation reaction mixture corresponding to 1% of the input for purification (Total) was also analyzed. Arrowheads indicate HRT1 or HRT1(30–290) (blue), HRBP or HRBP(55–257) (red), or MSP (gray). (**f**) Relative prenyltransferase activity of HRT1/HRBP-nanodisc and HRT1(30–290)/HRBP-nanodisc complexes. The raw data used to calculate relative activity are 12,220 ± 678 dpm for HRT1/HRBP and 160 ± 78 dpm for HRT1(30–290)/HRBP (means ± s.d. from three independent experiments).

We next attempted modeling for complexes formed by full-length cPT and cPTL proteins. The predicted structures (Supplementary Fig. S5) were similar to those of the corresponding complexes formed by NH_2_-terminally truncated cPTL (Fig. 5). The models for the full-length proteins showed that HRT1, HRT2, and PaCPT2 each possess an amphipathic α-helical domain in the NH_2_-terminal region (Fig. 7a–d and Supplementary Fig. S5), consistent with the membrane binding properties of these proteins. On the other hand, all identified cPTL family proteins of higher plants possess an NH_2_-terminal region that is predicted to serve as a transmembrane domain^8,30^. Given that the structure models do not reflect membrane assembly, the putative NH_2_-terminal transmembrane domains of HRBP and PaCBP are shown as horizontal α-helices in Fig. 7 and Supplementary Fig. S5.

To examine the function of the predicted NH_2_-terminal regions of HRT1 and HRBP, we prepared the truncated forms HRT1(30–290), which lacks the NH_2_-terminal amphipathic helix, and HRBP(55–257), which lacks the NH_2_-terminal region containing the putative transmembrane domain. The truncated proteins were co-expressed in the cell-free system in the presence of nanodiscs, and protein-nanodisc complexes were purified. SDS-PAGE analysis showed that, unlike the full-length proteins, HRT1(30–290) and HRBP(55–257) did not associate with the nanodiscs (Fig. 7e), suggesting that the deleted residues (1–29 of HRT1 and 1–54 of HRBP) are essential for interaction with the lipid bilayer. Co-expression of HRT1(30–290) and full-length HRBP revealed that a small amount of HRT1(30–290) was detected in the purified nanodisc complexes, whereas full-length HRT1 was not able to anchor HRBP(55–257) to the lipid membrane (Fig. 7e). The purified nanodiscs containing HRT1(30–290) and HRBP did not possess prenyltransferase activity (Fig. 7f).

## Discussion

We have shown here that the co-expression of HRT1 and HRBP of *H. brasiliensis* in the presence of nanodiscs reconstitutes prenyltransferase activity in vitro. As far as we are aware, this is the first demonstration that the combination of these proteins gives rise to such activity on a lipid-bilayer membrane. The order of sequential expression of HRT1 and HRBP did not affect enzyme activity, suggesting that each single subunit associated with nanodiscs is stable during purification and readily accepts the other subunit to form a functional prenyltransferase. On the other hand, a mixture of purified HRT1-nanodisc and HRBP-nanodisc complexes did not show substantial prenyltransferase activity, suggesting that the association of each protein with the lipid bilayer of different nanodiscs hinders interaction of HRT1 with HRBP and consequent reconstitution of prenyltransferase activity. Physical proximity of, and likely a direct interaction between, HRT1 and HRBP thus appears to be essential for prenyltransferase activity in vitro. Recent determination of the crystal structure of human DHDDS and NgBR revealed that these human cPT (DHDDS) and cPTL (NgBR) homologs form a heterodimeric complex^28,29^. The core enzyme of rubber synthesis may thus be similar to that of the dolichol biosynthetic enzymes of mammals that consist of cPT and cPTL subunits. We found that the predicted heterodimer models for plant cPT/cPTL complexes resembled the structure of the DHDDS/NgBR complex. Of note, amino acids at the putative contact interface are likely conserved among plant cPT/cPTL complexes and DHDDS/NgBR. Mutation of G252 or F253 of NgBR was previously found to hinder complex formation with DHDDS^28^. The conservation of these residues at the corresponding positions of plant cPTLs (Supplementary Fig. S6) suggests a key role for this region in cPT/cPTL complex formation.

Our in vitro analysis of HRT2 did not support functional relevance of this protein with regard to rubber biosynthesis. HRT2 mRNA has been found to be present at a high level in latex^31,32^, and HRT2 protein was detected in rubber particles by proteomics analysis^8^. In addition, polyisoprene synthesis activity was found to be conferred by heterologous expression of HRT2 in yeast^16^. On the other hand, we previously demonstrated the interaction between HRT1 and HRBP based on the yeast two-hybrid system^8^. However, no interaction between HRT2 and HRBP could be detected in the same system^8^. Combining our results, HRT2 seems non-functional in combination with HRBP. Mutation analysis of HRT2 compared to HRT1 amino acid sequence may clarify the functional difference between these homologous proteins. The possible role of HRT2 in NR biosynthesis thus requires further study.

We found that both HRT1-nanodisc and HRBP-nanodisc complexes could be used as a platform for the sequential assembly of HRT1/HRBP on nanodiscs and reconstitution of prenyltransferase activity. These results indicate that HRT1-nanodisc and HRBP-nanodisc complexes are stable. They also suggest that HRT1 and HRBP might form homodimers, given that structural studies for several prokaryotic cPT proteins^32–36^ and the yeast cPTL protein Nus1^37^ have revealed homodimer formation. We also generated similar homodimer models for HRT1 and HRBP using AlphaFold2 (Supplementary Fig. S7). Amino acid residues constituting the putative interfaces of cPT and cPTL homodimers are similar to those at the putative interface of cPT/cPTL heterodimers.

HRBP has been predicted to be a membrane-associated protein on the basis of the hydrophobicity of its NH_2_-terminal amino acid sequence and its similarity to higher plant homologs^8,30^. However, membrane association of HRT1 or HRT2 has not been clear. We were previously not able to determine the subcellular localization of HRT1 or HRT2 by heterologous expression in *Nicotiana benthamiana*, whereas co-expression with HRBP promoted the association of HRT1 or HRT2 with lipid membranes in these plant cells^8^. Heterologous expression of CPT6, an HRT2 homolog of *H. brasiliensis* Reyan 7–33-97, revealed it to be localized to the cytosol^38^. We here confirmed that HRT1 and HRBP each possess membrane binding activity, with such binding being stable during purification of protein-nanodisc complexes by IMAC. As mentioned above, the stability of membrane association of HRT1 and HRBP allowed the sequential reconstitution of the HRT1/HRBP complex on nanodiscs.

The structure models constructed in this study predicted the presence of an amphipathic α-helix at the NH_2_-termini of HRT1, HRT2, and PaCPT2, with this domain likely being responsible for membrane binding. We thus found that HRT1(30–290) alone did not bind to a lipid membrane. We also found that deletion of the NH_2_-terminal region of HRBP thought to contain a transmembrane domain resulted in the loss of affinity to lipid bilayers. Of interest, co-expression of full-length HRBP with HRT1(30–290) supported the association of the latter with nanodiscs, suggesting that HRBP inserted into a lipid membrane via its NH_2_-terminal domain may contribute to the stability of HRT1 binding to the membrane through physical interaction with HRBP, as has been proposed previously^8,38^. However, HRT1(30–290) did not show enzyme activity even when anchored to nanodiscs by co-expression with HRBP, suggesting that the interaction of HRT1 itself with the lipid membrane as well as with HRBP may be required for prenyltransferase activity.

We found that *P. argentatum* homologs of HRT1 and HRBP also reconstituted prenyltransferase activity on nanodiscs, with the combination of cPT and cPTL proteins being required for such reconstitution. All three PaCPT proteins of *P. argentatum* thus reconstituted prenyltransferase activity with the same partner protein, PaCBP. Based on RNA seq analysis, previous reports demonstrated that *PaCPT3* is predominantly expressed in stems and induced by cold stress^6,39^. Therefore, PaCPT3 has been considered as the main contributor to rubber biosynthesis. On the other hand, *PaCPT2* expression was found to be leaf specific, and *PaCPT1* expression was confirmed to be basal and ubiquitous^6,39^. Results in the report also indicated that cold shock also up-regulates expression of *PaCPT2* and *PaCBP*, but not that of *PaCPT1*. As well as in the previous report^6^, our study also demonstrated that PaCBP commonly reconstitutes polyisoprene synthesis activity with all three isoforms. However, we have to note that the translation efficiencies of the original mRNA sequences of the three isoforms might not be reflected in our biochemical analysis because we redesigned codon-optimized sequences for the synthesis of these proteins in vitro.

We also found that the prenyltransferase subunits of *H. brasiliensis* and *P. argentatum* were partially compatible, with HRBP being able to support the activity of PaCPT2. Amino acid sequence identities are 53% for HRT1 and PaCPT2, and 49% for HRBP and PaCBP. Although maximal activity was achieved with the subunit pairs from the same species, these results suggest that the distantly related plants *H. brasiliensis* and *P. argentatum* deploy similar heterosubunit enzymes for the biosynthesis of polyisoprenes. In addition, the higher activity of PaCPT2/PaCBP than HRT1/HRBP was constantly observed. The PaCPT2/PaCBP pair might form a more stable complex than the HRT1/HRBP pair. This difference may be due to the membrane environment of nanodiscs which have a flat lipid-bilayer.

Model structures were predicted for HRT2/HRBP, HRT1/PaCBP, and PaCPT2/HRBP heterodimers, even though only PaCPT2/HRBP manifested prenyltransferase activity in vitro. Thus, the results of structure prediction did not necessarily reflect those of the enzyme activity assays.

The reconstituted prenyltransferases in the present study did not generate NR-size polyisoprenes. Toluene-hexane extracts of none of the various reaction mixtures revealed incorporation of [^14^C]IPP into macromolecules (data not shown), possibly reflecting suboptimal conditions relating to the lipid environment or the lack of accessory proteins such as REF and SRPP^7,9,10,40^. We tested the co-expression of REF and SRPP together with HRT1 and HRBP in the presence of nanodiscs. However, because of the low affinity of REF and SRPP to nanodisc membranes, these proteins were lost during the purification step. The flat lipid bilayer structure of nanodiscs may not be favorable for interacting with these accessory proteins. Therefore, we could not evaluate the effects of REF and SRPP on HRT1/HRBP enzyme activity by the nanodisc system. A more suitable platform such as lipid monolayer vesicles may be utilized for clarifying the contributions of REF and SRPP to the enzyme activity. Further studies will thus be required to achieve in vitro reconstitution of the NR biosynthetic machinery.

## Methods

### Nanodisc preparation

A synthetic gene encoding the MSP derivative MSP1E3D1 with an NH_2_-terminal His_6_ tag^24^ was obtained from Eurofins Genomics. Expression and purification of MSP1E3D1 were performed as described previously^23^, with minor modifications. After bacterial cell culture, cells isolated by centrifugation were resuspended in a solution comprising 50 mM Tris–HCl (pH 7.5), 300 mM NaCl, and 20 mM imidazole. The cells were then subjected to extraction by ultrasonic treatment, the extract was centrifuged at 20,000 × *g* for 30 min at 4 °C, and the resultant supernatant was mixed with 2 ml of Ni–NTA agarose resin suspension (Qiagen) and incubated on a rotary shaker for 60 min at 4 °C before transfer of the mixture to a Poly-Prep Chromatography Column (Bio-Rad). The column bed was washed with 40 ml of a solution containing 50 mM Tris–HCl (pH 7.5), 300 mM NaCl, and 50 mM imidazole before elution with a solution containing 50 mM Tris–HCl (pH 7.5), 300 mM NaCl, and 250 mM imidazole. The eluted fractions were collected in 1.5-ml tubes, and those containing MSP1E3D1 were combined and subjected to dialysis (molecular size cutoff of 12 to 14 kDa) for 2 days at 25 °C with a 500-fold volume of a solution containing 50 mM Tris–HCl (pH 7.5) and 300 mM NaCl, with four changes of the dialysis buffer.

For nanodisc reconstitution, the MSP1E3D1 solution was mixed with an equal volume of 600 mM sodium cholate and agitated gently for 20 min at 25 °C and was then combined with other components to form a mixture containing 10 mM Tris–HCl (pH 8.0), 100 mM NaCl, 162 mM sodium cholate, 40 µM MSP1E3D1, and 2 mM asolectin (Sigma-Aldrich). The mixture was agitated gently for 60 min at 25 °C and then subjected to dialysis (molecular size cutoff of 12 to 14 kDa) for 2 days at 25 °C with a 500-fold volume of nanodisc buffer comprising 10 mM Tris–HCl (pH 8.0) and 100 mM NaCl, with four changes of the latter buffer. The mixture was centrifuged at 20,400 × *g* for 30 min at 4 °C, and the resulting supernatant was applied to a gel-filtration column of Superdex 200 Increase 10/300 GL (Cytiva). Chromatography was performed with nanodisc buffer at a flow rate of 0.5 ml/min and was monitored by measurement of absorbance at 280 nm, and the peak fractions corresponding to the assembled nanodiscs were collected. The eluate containing the nanodiscs was concentrated to 1 ml with the use of an Amicon Ultra-15 10 K device (Merck Millipore) and then mixed with 10 ml of translation buffer containing 30 mM HEPES–KOH (pH 7.8) and 100 mM potassium acetate before adjustment of the nanodisc concentration to 200 µM.

### Cell-free protein synthesis

Codon usage for nucleotide sequences of protein-coding regions were optimized for *Triticum aestivum* (wheat), and the DNA molecules were synthesized at Eurofins Genomics. GenBank accession numbers for the modified cDNAs are LC626733, LC626734, and LC626735 for HRT1, HRT2, and HRBP, respectively, of *H. brasiliensis*; LC626736, LC626737, LC626738, and LC626739 for PaCPT1, PaCPT2, PaCPT3, and PaCBP, respectively, of *P. argentatum*; and LC626740 for human β2AR. The synthesized sequences were cloned into the pYT08 vector^21^. Wheat-germ extract for cell-free protein synthesis (WEPRO 7240 or 7240H) was obtained from CellFree Sciences. Transcription and cell-free protein synthesis were performed as previously described^41^, with some modifications. We added nanodiscs to the translation reaction mixture at a final concentration of 15 μM. Procedural details are described in Supplementary Methods. For confirmation of guayule protein synthesis, [^14^C]Leu (0.185 kBq/μl) was added to the translation mixture and the purified protein-nanodisc complexes were subjected to SDS-PAGE and autoradiography as described previously^42^.

### Purification of protein-nanodisc complexes

Protein-nanodisc complexes were purified by IMAC based on the interaction of His_6_-tagged MSP1E3D1 with Ni–NTA agarose. The translation mixture was gently agitated in a microtube for 1 h at 4 °C with a fivefold volume of Ni–NTA agarose (Qiagen) that had been equilibrated with a solution containing 50 mM Tris–HCl (pH 7.5) and 300 mM NaCl. The mixture was then transferred to a Poly-Prep Chromatography Column (Bio-Rad), the column was washed with three column-bed volumes of a solution containing 50 mM Tris–HCl (pH 7.5), 300 mM NaCl, and 50 mM imidazole, and the protein-nanodisc complexes were eluted with a solution containing 50 mM Tris–HCl (pH 7.5), 300 mM NaCl, and 250 mM imidazole and then concentrated to ~ 50 µl by centrifugation in a VIVASPIN 500 tube (Sartorius), during which the buffer was exchanged with 50 mM Tris–HCl (pH 7.5). The protein-nanodisc complexes were denatured by incubation in the presence of 2% SDS and 5% 2-mercaptoethanol for 1 h at 37 °C for confirmation of their composition by SDS-PAGE on a 12% acrylamide gel. The gel was stained with CBB (Bio Craft), and the concentration of the purified protein in the samples was estimated relative to that of bovine serum albumin (BSA) standard solutions. Band intensity was calculated with the use of ImageJ software^43^ (Supplementary Fig. S2). Given that the molecular sizes of PaCPT1 and MSP are similar and that their bands overlapped in CBB-stained gels, the ratio of PaCPT1 to MSP was estimated on the basis of the relative ratio of PaCPT2 to MSP in the corresponding complex.

### Measurement of prenyltransferase activity

The prenyltransferase activity of protein-nanodisc complexes was measured with a modified version of a previously described method^8^. Purified protein-nanodisc complexes were added to a reaction mixture (final volume of 50 µl) containing 50 mM Tris–HCl (pH 7.5), 2 mM dithiothreitol, 4 mM MgCl_2_, 15 µM farnesyl pyrophosphate, and 125 nCi of [4-^14^C]IPP (Perkin Elmer, 50.6 mCi/mmol) in deionized water. The amounts of protein-nanodisc complexes added to the enzyme assay were adjusted so that 0.5 µg of HRT1, HRT2, PaCPT1, PaCPT2, or PaCPT3 was contained in each reaction mixture. For protein-nanodisc complexes without HRT or PaCPT proteins, the amounts were adjusted so that 0.5 µg of HRBP or PaCBP was contained in each reaction mixture. To confirm that the nanodiscs did not show cPT enzyme activity as a result of nonspecific transfer of proteins from the translation reaction, those assembled with β2AR were used as a negative control. The reaction mixtures were incubated for 20 h at 16 °C, after which 100 µl of deionized water saturated with NaCl were added and polyisoprenes with a common chain length shorter than that of NR were extracted with 500 µl of 1-butanol that had been saturated with NaCl-saturated water. Any NR-size polyisoprenes in the remaining aqueous layer were extracted with 500 µl of toluene/hexane (1:1, v/v). The amount of radioactivity in the extracts was measured with a liquid scintillation counter. The enzyme assay was performed in triplicate, and background counts were measured for reaction mixtures without protein-nanodisc complexes according to the same procedure. The background value was subtracted from each experimental value to determine the counts for [^14^C]IPP incorporation. For comparison of the enzyme activities of full-length PaCPT3 and PaCPT3_dN15, or of full-length HRT1 and HRT1(30–290), 12.5 nCi of [^14^C]IPP (5 mCi/mmol) was added to the reaction mixture.

### Reversed-phase TLC analysis

Analysis of chain length for polyisoprenes extracted by 1-butanol was performed as previously described^16^. The extracts were hydrolyzed with acid phosphatase (Sigma) to produce alcohols, which were then extracted with pentane for analysis by reversed-phase TLC with HPTLC Silica Gel 60 RP-18 (Merck) and an acetone/water (39:1, v/v) solvent system. The [^14^C]IPP polymers spread on the TLC plate were detected with a FLA7000 instrument (Fujifilm). The chain length of the reaction products was estimated with the use of carbon number–defined *Z,E*-mixed polyprenols (C_55_, C_60_, C_85_, and C_90_) as authentic standards, as described previously^16^.

### Model building for protein complexes

Construction of structure models for protein complexes was performed on Google Colaboratory with the use of AlphaFold2_complexes^27^, which is a modified form of AlphaFold version 2.0^26^. Structure models were generated in five patterns for combinations of full-length HRT1, HRT2, or PaCPT2 with full-length HRBP or PaCBP or truncation mutants thereof lacking a putative NH_2_-terminal transmembrane region—HRBP(55–257) and PaCBP(53–250), respectively (Supplementary Table S1). Molecular superposition and image generation were performed with PyMOL version 2.5.1 (Schrodinger). The putative NH_2_-terminal amphipathic α-helical regions of HRT1 and PaCPT2 were colored according to the hydrophobic scale of the amino acid residues^44^ (Fig. 7a,b).

### Immunoblot analysis

After SDS-PAGE, separated proteins were transferred to a polyvinylidene difluoride membrane (pore size, 0.45 µm) for immunoblot analysis. For detection of HRT1 and HRBP, polyclonal antibodies were custom-generated by Eurofins Genomics by injection of rabbits with conjugated peptides containing specific amino acid sequences for HRT1/HRT2 (NH_2_-C + FAKKHKLPEGGGHK-COOH) or HRBP (NH_2_-C + YDSKGVLKTNK-COOH and NH_2_-C + EAVEKDVLLDQKQM-COOH), respectively, as previously described^8^. Immune complexes were detected with horseradish peroxidase–conjugated goat antibodies to rabbit immunoglobulin G (Proteintech) and ImmunoStar Zeta (FUJIFILM Wako Pure Chemical Corporation). For detection of His_6_-tagged MSP1E3D1, a mouse monoclonal antibody to the His_6_ tag (Wako) and horseradish peroxidase–conjugated sheep antibodies to mouse immunoglobulin G (Jackson Research Laboratory) were used.

## Supplementary Information

Supplementary Information.

## Data Availability

The datasets generated during and/or analyzed during the current study are available from the corresponding author upon reasonable request.
