# Supplementary material for: Reconstitution of prenyltransferase activity on nanodiscs by components of the rubber synthesis machinery of the Para rubber tree and guayule
Source: Sci Rep. 2022 Mar 8;12:3734. doi: 10.1038/s41598-022-07564-y (PMC8904820; doi:10.1038/s41598-022-07564-y)

Supplementary Information

Supplementary Methods

Cell-free protein synthesis with wheat-germ extract. Template mRNAs for cell-free protein synthesis were produced by in vitro transcription with SP6 RNA polymerase. Nucleotide sequences for the recombinant proteins were optimized for the wheat-germ cell-free system and cloned into the pYT08 vector. Plasmids were amplified in *E. coli*, isolated with the use of a Plasmid Maxi Kit (Qiagen), and then subjected to in vitro transcription in a transcription mixture of 100 µl containing 10 µg of plasmid as template DNA, 40 mM Tris-HCl (pH 7.5), 6 mM $MgCl_2$, 2 mM spermidine, 10 mM dithiothreitol, 0.01% BSA, 3 mM of each nucleoside triphosphate, RNase inhibitor (0.8 U/µl) (Promega), and SP6 RNA polymerase (0.8 U/µl) (Promega). The transcription mixture was gently mixed by pipetting and incubated at 37°C for 3 h in a water bath. The mixture was then centrifuged at 20,000 × *g* for 1 min, and the resulting supernatant was transferred to a new microtube, mixed with 13 µl of 7.5 M ammonium acetate and 250 µl of 100% ethanol, and incubated at –20°C for 15 min. The synthesized mRNAs were isolated by centrifugation at 20,000 × *g* for 20 min at 4°C, and the resulting pellet was washed with 800 µl of 70% ethanol, isolated again by centrifugation, allowed to dry in air, and then dissolved in 21 µl of deionized water.

The translation mixture (total volume of 50 µl) contained 30 mM HEPES-KOH (pH 7.8), 100 mM potassium acetate, 2.7 mM magnesium acetate, 0.4 mM spermidine, 2.5 mM dithiothreitol, 0.3 mM of each amino acid, 1.2 mM ATP, 0.25 mM GTP, 16 mM creatine phosphate, creatine kinase (0.4 mg/ml), RNase inhibitor (0.8 U/µl), 15 µM asolectin nanodiscs, 15 µl of wheat-germ extract (CellFree Sciences), and 100 µg of mRNA (in the case of co-expression of two different proteins, 50 µg of each mRNA were added). The reaction mixture was placed in a dialysis cup with a molecular size cutoff of 12 kDa (Biotech International) and dialyzed against 1 ml of substrate supply solution containing 30 mM HEPES-KOH (pH 7.8), 100 mM potassium acetate, 2.7 mM magnesium acetate, 0.4 mM spermidine, 2.5 mM dithiothreitol, 0.3 mM of each amino acid, 1.2 mM ATP, 0.25 mM GTP, 16 mM creatine phosphate, and 0.005% NaN_3. The mixture was then incubated at 16°C for 48 h.

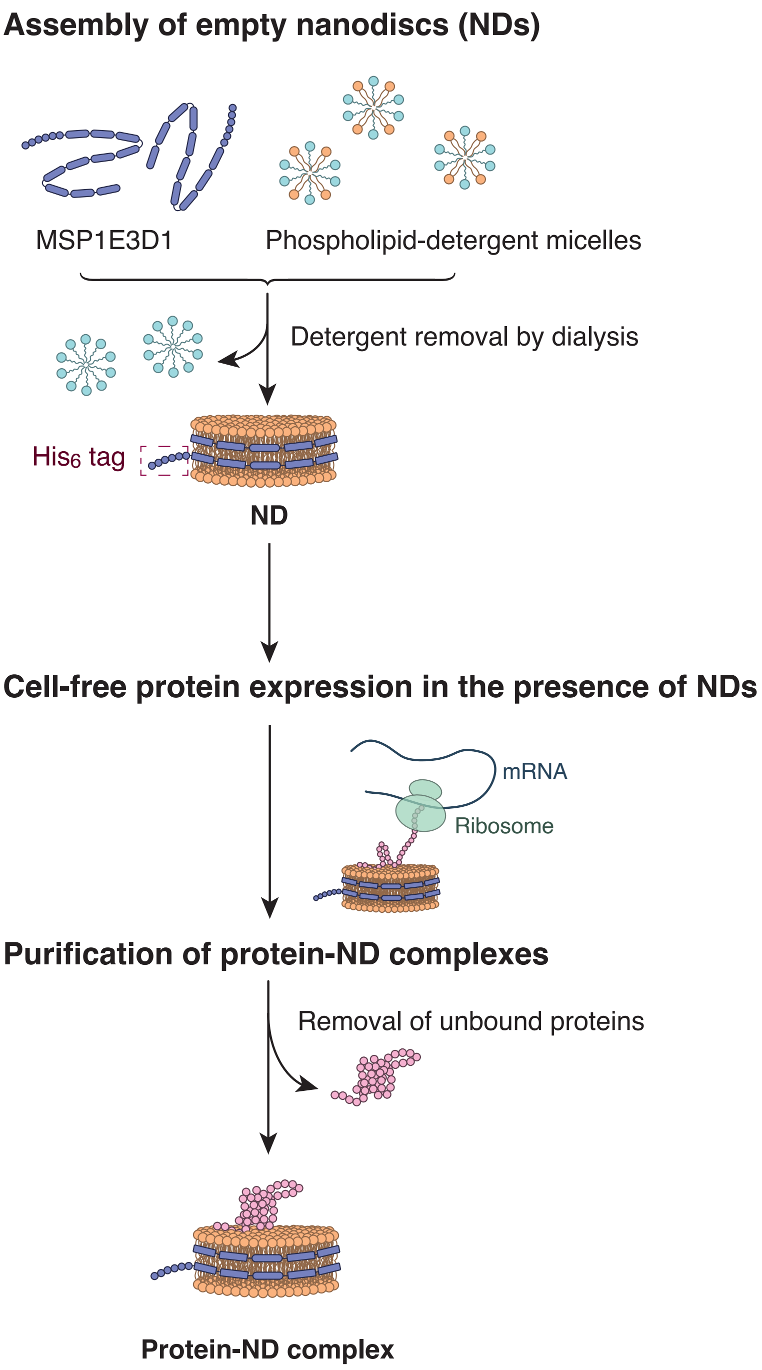

Supplementary Figure S1. Procedure for the preparation of protein-nanodisc complexes. Empty nanodiscs (NDs) were prepared from purified membrane scaffold protein (MSP1E3D1) and asolectin, and were then purified by IMAC in a manner dependent on the NH_2-terminal His_6 tag of MSP. The purified nanodiscs were then added to a cell-free translation system, and co-translationally assembled (or bound) proteins were co-purified with the nanodiscs by IMAC.

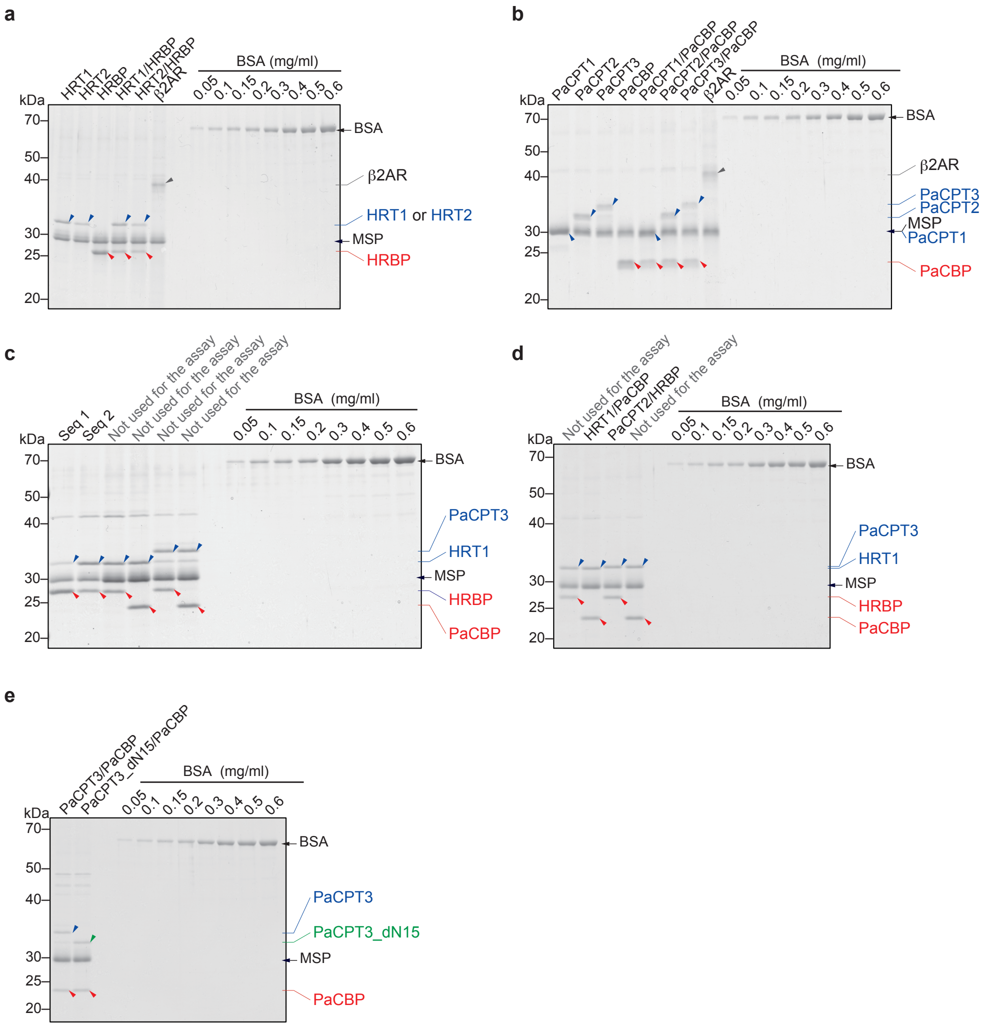

Supplementary Figure S2. Estimation of protein concentration for protein-nanodisc complexes by SDS-PAGE and CBB staining and comparison with a BSA standard. *Hevea brasiliensis* (HRT1, HRT2, and HRBP) and *P. argentatum* (PaCPT1, PaCPT2, PaCPT3, PaCPT3_dN15, and PaCBP) proteins as well as human β2AR were assembled with asolectin nanodiscs by cell-free protein synthesis with a wheat-germ translation system. The resulting protein-nanodisc complexes were purified by Ni-NTA column chromatography and then subjected to SDS-PAGE and CBB staining together with BSA standard solutions. The protein concentration of each sample was estimated from band intensity with ImageJ software. The samples shown here include those used for prenyltransferase activity assays in Figure 1 (**a**), Figure 3 (**b**), Figure 2 (**c**), Figure 4 (**d**), and Supplementary Figure S4 (**e**). Some of the samples were not used for the enzyme assay.

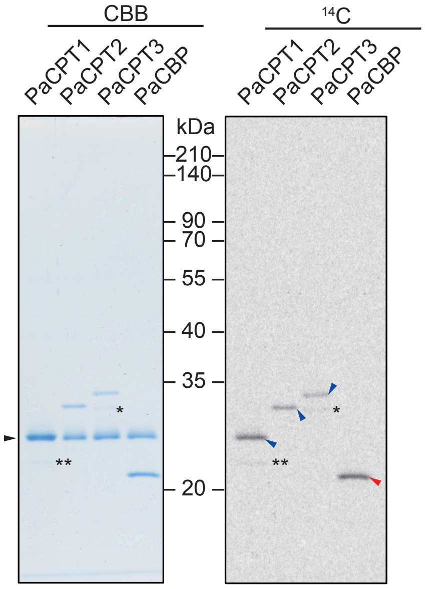

Supplementary Figure S3. Analysis of additional products in cell-free translation reactions. PaCPT1, PaCPT2, and PaCPT3 were synthesized in a cell-free system in the presence of nanodiscs and [^{14}C]leucine. The purified protein-nanodisc complexes were then subjected to SDS-PAGE, and the gel was stained with CBB (left panel) and subjected to autoradiography (right panel). Single and double asterisks indicate minor products in the PaCPT3 and PaCPT1 reaction mixtures, respectively. The black arrowhead indicates the band for MSP of the nanodiscs, which overlaps with that for PaCPT1. Blue and red arrowheads indicate the major ^{14}C-labeled proteins, whose apparent molecular sizes reflect their predicted molecular masses of 31.3 kDa for PaCPT1, 33.2 kDa for PaCPT2, and 34.8 kDa for PaCPT3 and of 28.7 kDa for PaCBP, respectively.

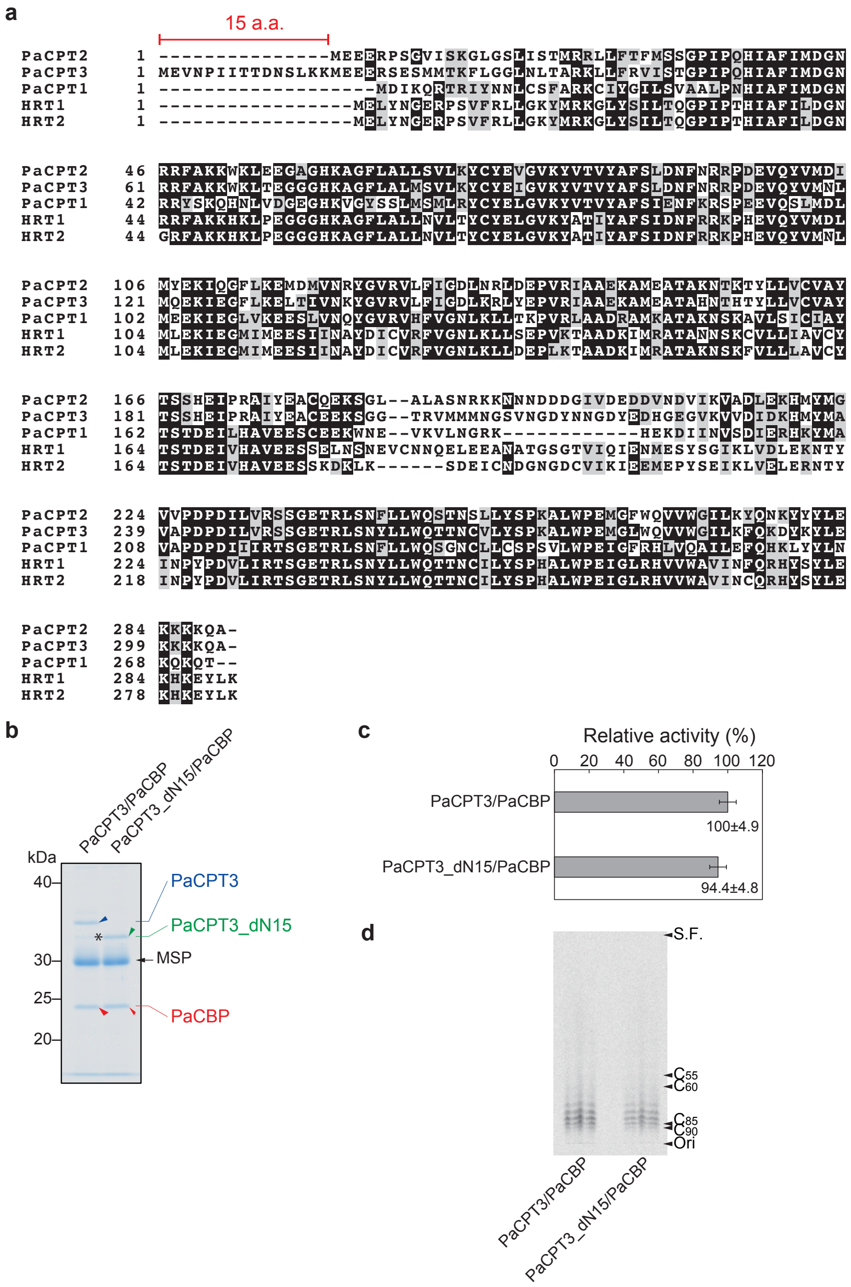

Supplementary Figure S4. Analysis of the truncated form of PaCPT3. (**a**) Alignment of the predicted amino acid sequences of HRT1, HRT2, PaCPT1, PaCPT2, and PaCPT3. Hyphens indicate gaps introduced to optimize alignment. Identical or similar residues among the five proteins are indicated by dark and light shading, respectively, and residue numbers are shown on the left of the sequences. PaCPT2 and PaCPT3 are closely related, sharing the highest sequence identity (89%) among the five proteins. PaCPT3 possesses a methionine at position 16 that corresponds to the first methionine of PaCPT2. (**b**) PaCBP as well as either the truncated form of PaCPT3 lacking the NH_2-terminal 15 amino acids (PaCPT3_dN15) or the

wild-type protein were synthesized in a cell-free system in the presence of nanodiscs, and the purified protein-nanodisc complexes were characterized by SDS-PAGE and CBB staining. The asterisk indicates a protein in the PaCPT3 reaction mixture with a mobility similar to that of PaCPT3_dN15. (**c**) Relative prenyltransferase activity of PaCPT3/PaCBP-nanodisc and PaCPT3_dN15/PaCBP-nanodisc complexes. The raw data used to calculate relative activity are 22,683 ± 1105 dpm for PaCPT3/PaCBP and 21,406 ± 1096 dpm for PaCPT3_dN15/PaCBP (means ± s.d. from three independent experiments). (**d**) TLC analysis of extracts prepared from enzyme assay mixtures as in (**c**) with 1-butanol. Both the PaCPT3/PaCBP and PaCPT3_dN15/PaCBP complexes showed similar patterns of polyisoprene chain length.

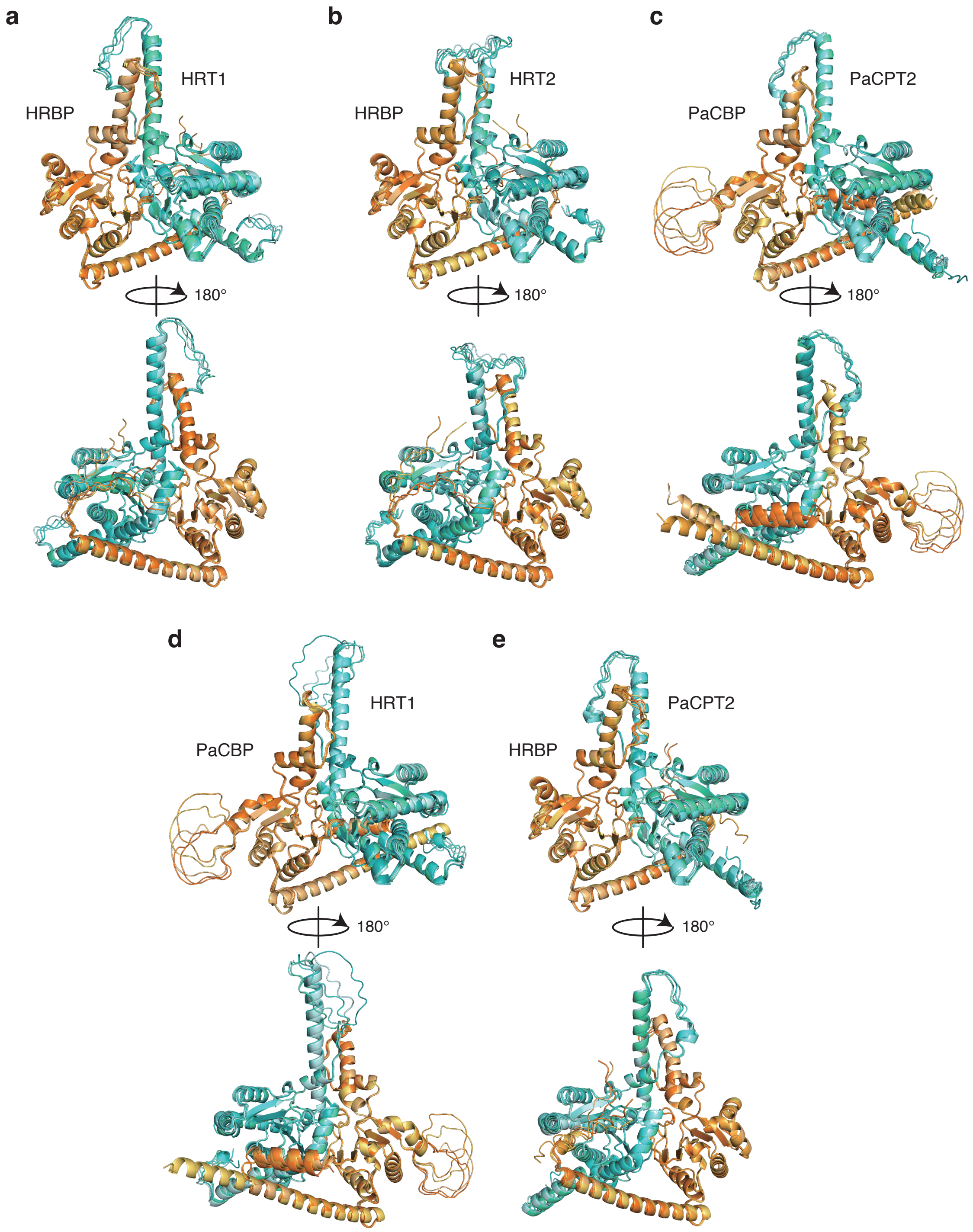

Supplementary Figure S5. Structural models of full-length CPT/CPTL complexes. HRT1/HRBP (**a**), HRT2/HRBP (**b**), PaCPT2/PaCBP (**c**), HRT1/PaCBP (**d**), and PaCPT2/HRBP (**e**) complexes are shown, with cPT and cPTL proteins colored cyan orange, respectively. Each pair of images represents views before and after vertical rotation through 180 degrees.

cPT

```
HRT1        --MELYNGERPSVFRLLGKYMRKGLYSILTQGPIPTHIAFILDGNRRFAKKHKLPEGGGH 58
PaCPT2      MEEERPSGVISKGLGSLISTMRRLLFTFMSSGPIPQHIAFIMDGNRRFAKKWKLEEGAGH 60
DHDDS       ---------MSWIKEGELSLWERFCANIIKAGPMPKHIAFIMDGNRRYAKKCQVERQEGH 51
                      .       .  .:   .::. **:* *****:*****:*** :: .  **

HRT1        KAGFLALLNVLTYCYELGVKYATIYAFSIDNFRRKPHEVQYVMDLMLEKIEGMIMEESII 118
PaCPT2      KAGFLALLSVLKYCYEVGVKYVTVYAFSLDNFNRRPDEVQYVMDIMYEKIQGFLKEMDMV 120
DHDDS       SQGFNKLAETLRWCLNLGILEVTVYAFSIENFKRSKSEVDGLMDLARQKFSRLMEEKEKL 111
            . **  * ..* :* ::*:  .*:****::**.*   **: :**:  :*:. :: * . :

HRT1        NAYDICVRFVGNLKLLSEPVKTAADKIMRATANNSKCVLLIAVCYTSTDEIVHAVEESSE 178
PaCPT2      NRYGVRVLFIGDLNRLDEPVRIAAEKAMEATAKNTKTYLLVCVAYTSSHEIPRAIYEACQ 180
DHDDS       QKHGVCIRVLGDLHLLPLDLQELIAQAVQATKNYNKCFLNVCFAYTSRHEISNAVREMAW 171
            : :.: : .:*:*: *   ::    : :.** : .*  * :...*** .** .*: * .

HRT1        LNSNEVCNNQELEEANATGSGTVIQIENMESYSGIKLVDLEKNTYINPYPDVLIRTSGET 238
PaCPT2      EKSGLALASNRKKNNNDDDG--IVDEDDVNDVIKVADLEKHMYMGVVPDPDILVRSSGET 238
DHDDS       GVEQGLLDPSDISES--------------------LLDKCLYTNRSPHPDILIRTSGEV 210
              .      .  .:                    ::        * **:*:*:***.

HRT1        RLSNYLLWQTTNCILYSPHALWPEIGLRHVVWAVINCQRHYSYLEKHKEYLK-------- 290
PaCPT2      RLSNFLLWQSTNSLLYSPKALWPEMGFWQVVWGILKYQNKYYYLEKKKKQA--------- 289
DHDDS       RLSDFLLWQTSHSCLVFQPVLWPEYTFWNLFEAILQFQMNHSMLQKARDMYAEERKRQQL 270
            ***::****:::. *    .****  : ::. .::: * ::  *:* :.

HRT1        ------------------------------------------------------------
PaCPT2      ------------------------------------------------------------
DHDDS       ERDQATVTEQLLREGLQASGDAQLRRTRLHKLSARREERVQGFLQALELKRADWLARLGT 330


HRT1        ---
PaCPT2      ---
DHDDS       ASA 333
```

cPTL

```
HRBP        MDLKPGAGGQRVNRLVDPISYHFLQFLWRTLHLLVSLWYLQVSMVQMIEGFLISSGLVKR 60
PaCBP       --MDLVAESQKFFRRTSQSGSIVLFLLWHVVHLTISVLYIVREIFRAIESYLITNGYVKT 58
NgBR        ---MTGLYELVWRVLHALLCLHRTLTSWLRVRFGTWNWIWRRCCRAASAAVLAPLGFTLR 57
                                     *  :::               . * . * .

HRBP        YG--------------------------------ALDIDKVRYLAIVVDSEEAYQISKV 87
PaCBP       YT--------------------------------NINLQRVKYLGIVVDSDEARNISKV 85
NgBR        KPPAVGRNRRHHRHPRGGSCLAAAHHRMRWRADGRSLEKLPVHMGLVITEVEQEPSFSDI 117
                                               ::   *:   ::.: ::  .:*.:

HRBP        IQLLKWVEDMGVKHLCLYDSKGVLKTNKKTIMESLNN-------------AMPFEEAVEK 134
PaCBP       VELLEWLSAIGVKKICLYDREGVLKKSKAVIMERFGS-------------TETSNDSAVA 132
NgBR        ASLVVWCMAVGISYISVYDHQGIFKRNNSRLMDEILKQQQELLGLDCSKYSPEFANSNDK 177
             .*: *   :*:. :.:** :*::* .:  :*: : .             :    ::

HRBP        DVLLDQKQMTVEFASSSDGKEAITRAANVLFMKYLKYAKTGVGKEEPCFTEDQMDEALKA 194
PaCBP       NPLS-KKRMDFEFVSITDGKEAVAKAANLLFKKYYVDEDS----EKPFFTETHLTEALKT 187
NgBR        DDQVLNCHLAVKVLSPEDGKADIVRAAQDFCQLVAQKQKR-----PTDLDVDTLASLLSS 232
            :    : :: .:. *  ***  :.:**: :         .       . :    : . *.:

HRBP        IGYKGPEPDLLLIYGPVRCHLGFSPWRLRYTEMVHMGPLRYMNLGSLKKAIHRFTTVQQN 254
PaCBP       LGQVEPDPDLLLIYGPVRCHLGFPAWRLRYTEMVHMGPLKYKKFGLILKAIHRFTKVKQN 247
NgBR        NG--CPDPDLVLKFGPVDSTLGFLPWHIRLTEIVSLPSHLNISYEDFFSALRQYAACEQR 290
             *   *:***:* :*** . *** .*::* **:* : .    .   : .*::::  :*.

HRBP        YGT 257
PaCBP       YGS 250
NgBR        LGK 293
             *.
```

Supplementary Figure S6. Alignment of CPT (HRT1, PaCPT2, and DHDDS) and CPTL (HRBP, PaCBP, and NgBR) protein sequences. Alignment was performed with the CLUSTALW algorithm. Red boxes indicate amino acids at the interface of the DHDDS/NgBR complex and the predicted interfaces of HRT1/HRBP and PaCPT2/PaCBP (Figure 6). Hyphens indicate gaps introduced to optimize alignment, and periods, colons, and asterisks indicate conservation between groups of weakly similar properties, conservation between groups of strongly similar properties, and positions which have a single, fully conserved residue, respectively.

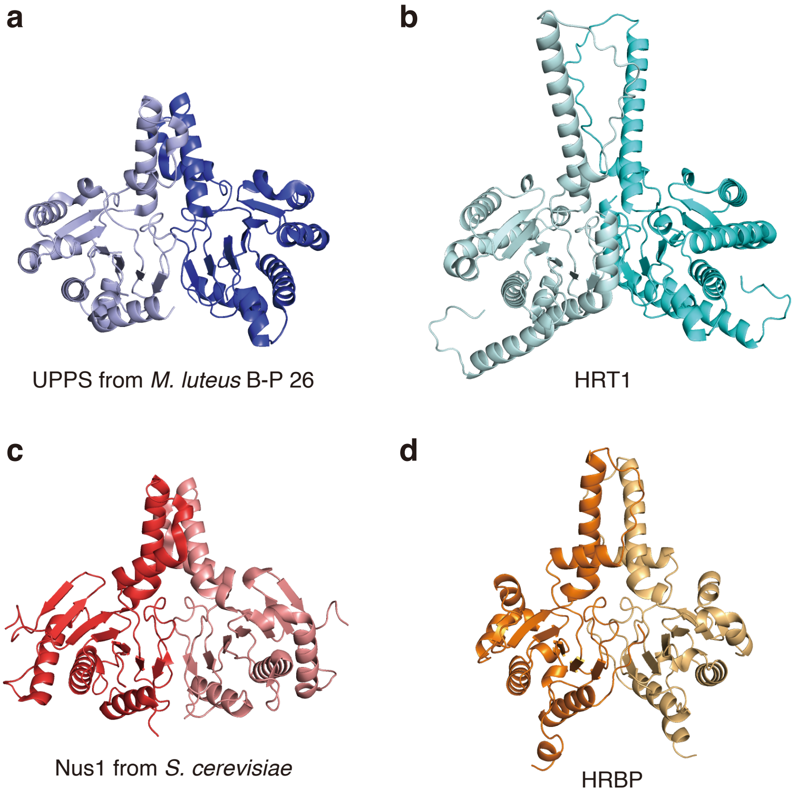

Supplementary Figure S7. Structure of the undecaprenyl diphosphate synthase (UPPS) homodimer from *Micrococcus luteus* B-P 26 (PDB code 1F75) (**a**), structural model of an HRT1 homodimer (**b**), structure of Nus1 from *Saccharomyces cerevisiae* (PDB code 6JCN) (**c**), and structural model of an HRBP homodimer (**d**) generated by AlphaFold2.

Supplementary Table S1. Amino acid sequences used as input for AlphaFold2 modeling.

Protein	Amino acid sequence
HRT1	MELYNGERPSVFRLLGKYMRKGLYSILTQGPIPTHIAFILDGNRRFAKKHKLPE GGGHKAGFLALLNVLTYCYELGVKYATIYAFSIDNFRRKPHEVQYVMDLMLE KIEGMIMEESIINAYDICVRFVGNLKLLSEPVKTAADKIMRATANNSKCVLLIA VCYTSTDEIVHAVEESSELNSNEVCNNQELEEANATGSGTVIQIENMESYSGIK LVDLEKNTYINPYPDVLIRTSGETRLSNYLLWQTTNCILYSPHALWPEIGLRHV VWAVINCQRHYSYLEKHKEYLK
HRT2	MELYNGERPSVFRLLGKYMRKGLYSILTQGPIPTHIAFILDGNGRFAKKHKLPE GGGHKAGFLALLNVLTYCYELGVKYATIYAFSIDNFRRKPHEVQYVMNLMLE KIEGMIMEESIINAYDICVRFVGNLKLLDEPLKTAADKIMRATAKNSKFVLLLA VCYTSTDEIVHAVEESSKDKLKSDEICNDGNGDCVIKIEEMEPYSEIKLVELER NTYINPYPDVLIRTSGETRLSNYLLWQTTNCILYSPHALWPEIGLRHVVWAVIN CQRHYSYLEKHKEYLK
PaCPT2	MEEERPSGVISKGLGSLISTMRRLLFTFMSSGPIPQHIAFIMDGNRRFAKKWKLE EGAGHKAGFLALLSVLKYCYEVGVKYVTVYAFSLDNFNRRPDEVQYVMDIM YEKIQGFLKEMDMVNRYGVRVLFIGDLNRLDEPVRIAAEKAMEATAKNTKTY LLVCVAYTSSHEIPRAIYEACQEKSGLALASNRKKNNNDDDGIVDEDDVNDVI KVADLEKHMYMGVVPDPDILVRSSGETRLSNFLLWQSTNSLLYSPKALWPEMG FWQVVWGILKYQNKYYYLEKKKKQA
HRBP	MDLKPGAGGQRVNRLVDPISYHFLQFLWRTLHLLVSLWYLQVSMVQMIEGFL ISSGLVKRYGALDIDKVRYLAIVVDSEEAYQISKVIQLLKWVEDMGVKHLCLY DSKGVLKTNKKTIMESLNNAMPFEEAVEKDVLLDQKQMTVEFASSSDGKEAI TRAANVLFMKYLKYAKTGVGKEEPCFTEDQMDEALKAIGYKGPEPDLLLIYG PVRCHLGFSPWRLRYTEMVHMGPLRYMNLGSLKKAIHRFTTVQQNYGT
HRBP(55–257)	SGLVKRYGALDIDKVRYLAIVVDSEEAYQISKVIQLLKWVEDMGVKHLCLYD SKGVLKTNKKTIMESLNNAMPFEEAVEKDVLLDQKQMTVEFASSSDGKEAIT RAANVLFMKYLKYAKTGVGKEEPCFTEDQMDEALKAIGYKGPEPDLLLIYGP VRCHLGFSPWRLRYTEMVHMGPLRYMNLGSLKKAIHRFTTVQQNYGT
PaCBP	MDLVAESQKFFRRTSQSGSIVLFLLWHVVHLTISVLYIVREIFRAIESYLITNGYV KTYTNINLQRVKYLGIVVDSDEARNISKVVELLEWLSAIGVKKICLYDREGVLK KSKAVIMERFGSTETSNDSAVANPLSKKRMDFEFVSITDGKEAVAKAANLLFKK YYVDEDSEKPFFTETHLTEALKTLGQVEPDPDLLLIYGPVRCHLGFPAWRLRYT EMVHMGPLKYKKFGLILKAIHRFTKVKQNYGS

PaCBP(53–250)	NGYVKTYTNINLQRVKYLGIVVDSDEARNISKVVELLEWLSAIGVKKICLYDR<br>EGVLKKSKAVIMERFGSTETSNDSAVANPLSKKRMDFEFVSITDGKEAVAKAAN<br>LLFKKYYVDEDSEKPFFTETHLTEALKTLGQVEPDPDLLLIYGPVRCHLGFPAW<br>RLRYTEMVHMGPLKYKKFGLILKAIHRFTKVKQNYGS

Original images of gels and blots

The area surrounded by the dashed line is cropped and shown as figures in this paper.

Figure 1a

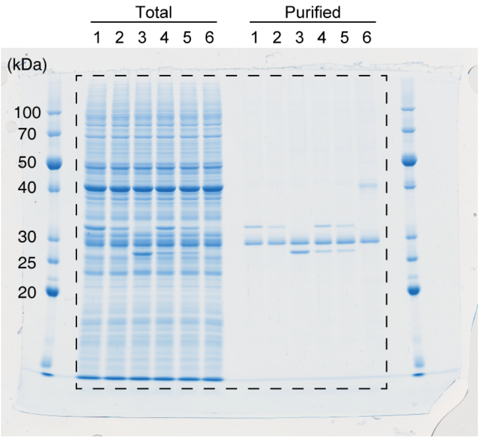

Figure 2b

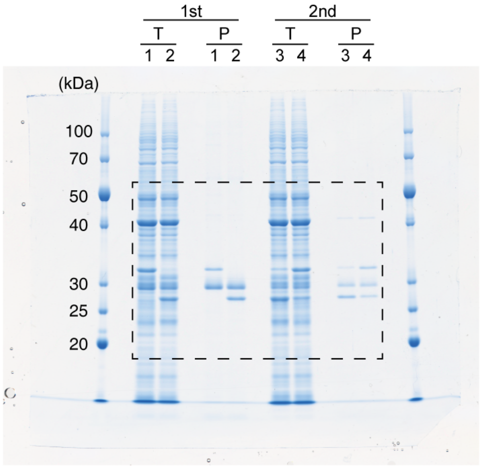

Figure 3a

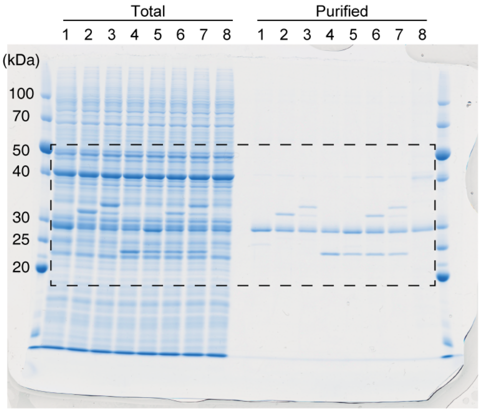

Figure 4a

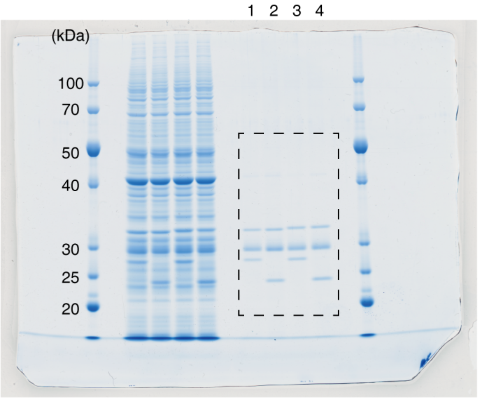

Figure 7e

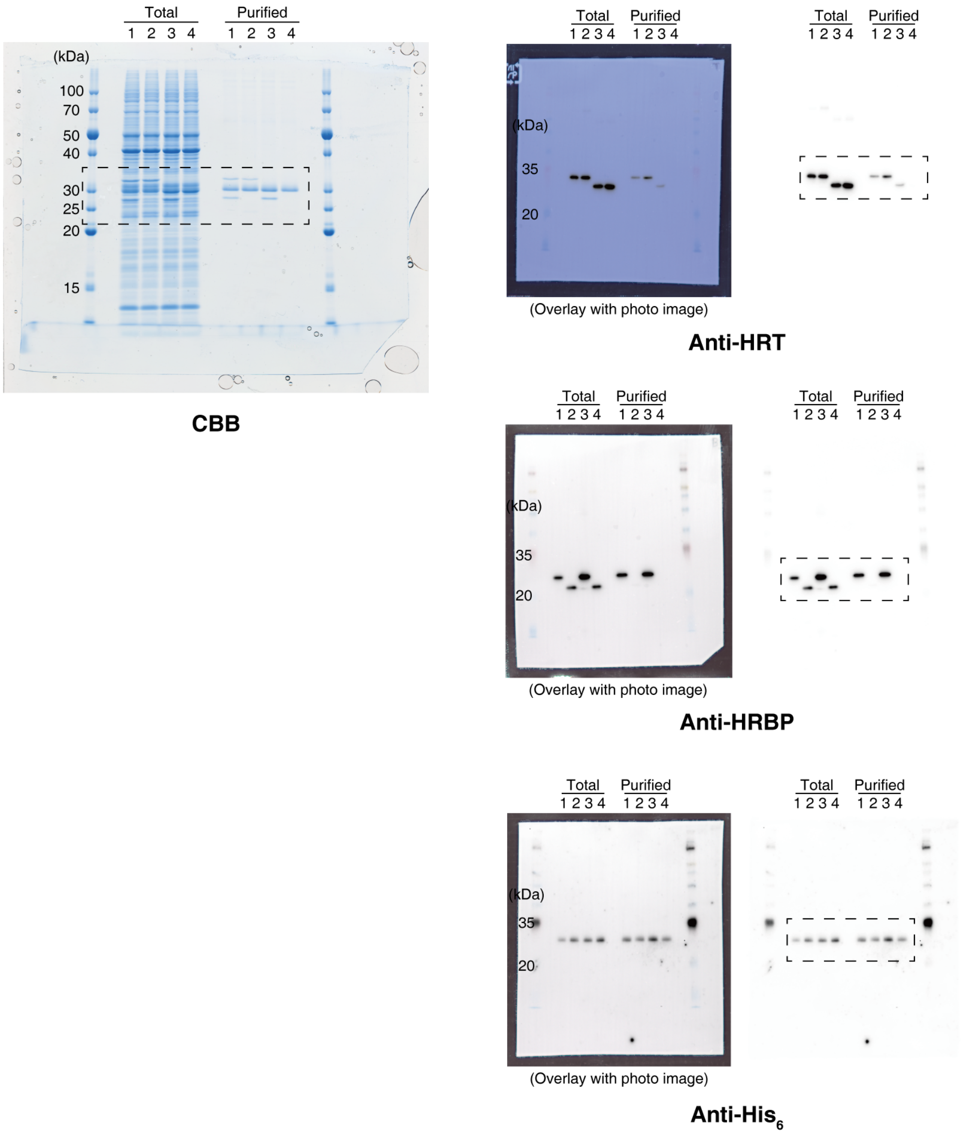
Total
Purified
1 2 3 4
(kDa)
100
70
50
40
30
25
20
15
CBB
(Overlay with photo image)
Anti-HRT
Anti-HRBP
Anti-His6
35
20

Supplementary Figure S2

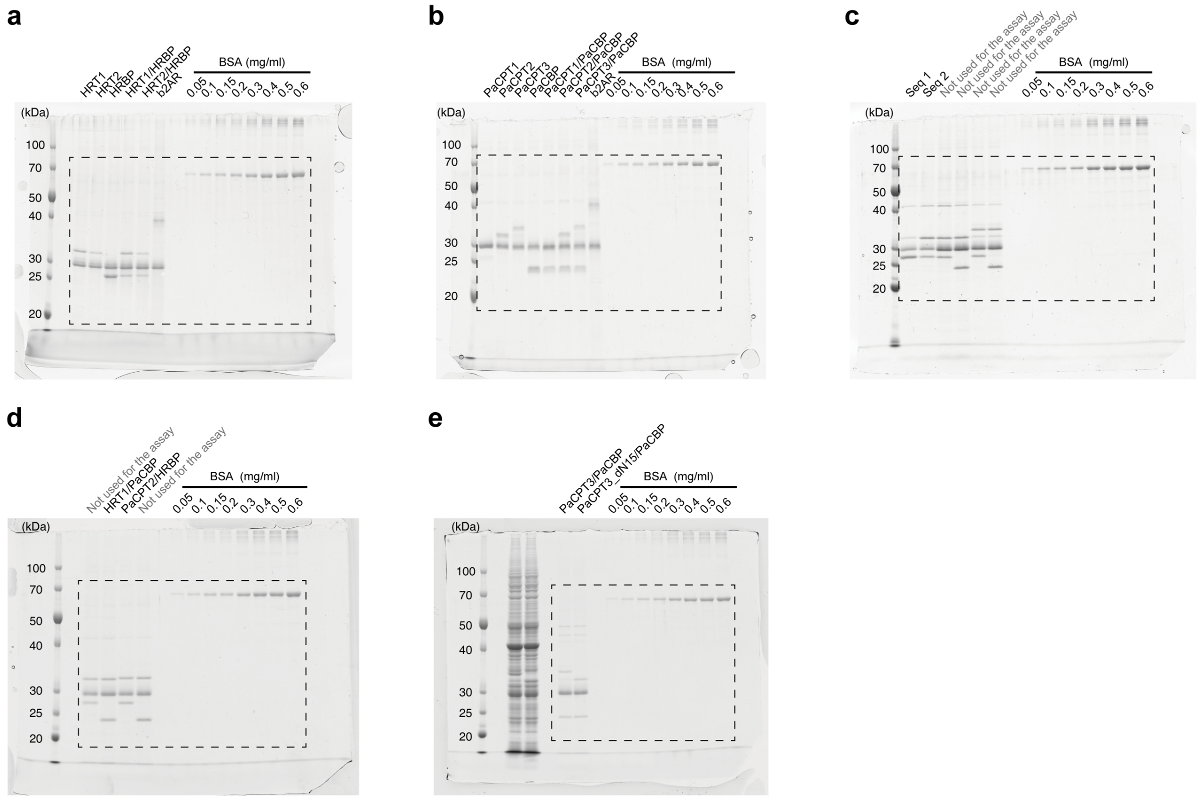

Supplementary Figure S3

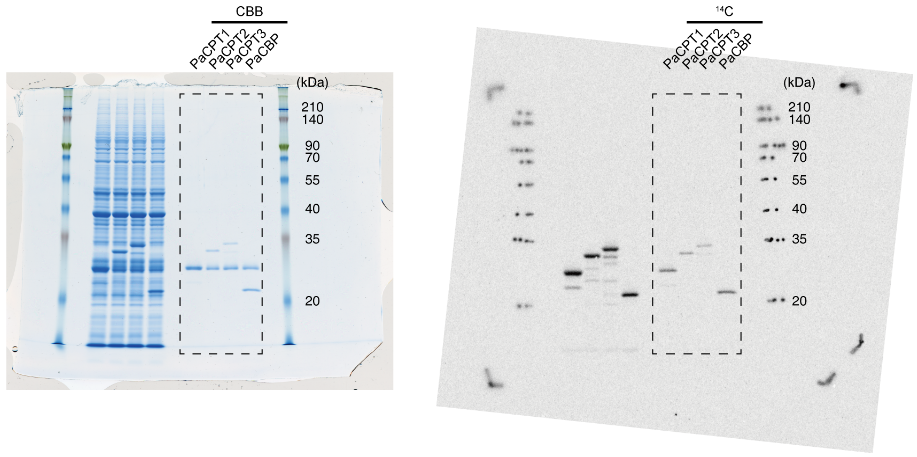

Supplementary Figure S4b

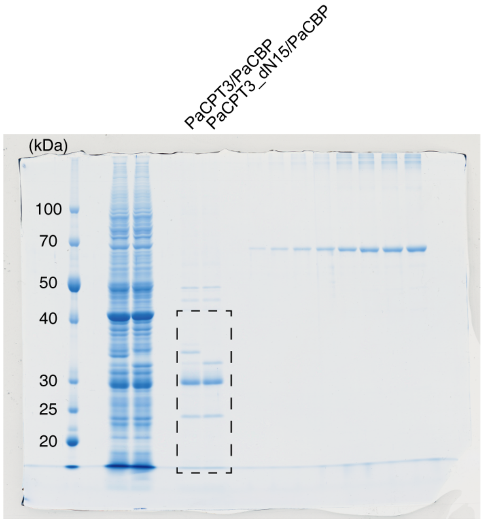

Supplement: Supplementary file 1 — Supplementary Information. [file 41598_2022_7564_MOESM1_ESM.pdf]
